# The illusion of meritocracy: addressing systemic gender gaps in bioinformatics

**DOI:** 10.3389/fbinf.2026.1891103

**Published:** 2026-09-11

**Authors:** Aldana A. Cepeda Dean, Natalia Rego, Luisa Berná

**Affiliations:** 1 Instituto de Investigaciones Biotecnológicas (IIBio), Universidad Nacional de San Martín (UNSAM), and Consejo Nacional de Investigaciones Científicas y Técnicas (CONICET), Buenos Aires, Argentina; 2 Escuela de Bio y Nanotecnologías (EByN), UNSAM, Buenos Aires, Argentina; 3 Bioinformatics Unit, Institut Pasteur de Montevideo, Montevideo, Uruguay; 4 Laboratorio de Genómica Evolutiva, Facultad de Ciencias, Universidad de la República, Montevideo, Uruguay; 5 Laboratory of Apicomplexan Biology, Institut Pasteur de Montevideo, Montevideo, Uruguay

**Keywords:** bioinformatics & compuational biology, citation gap, gender inequality, the Matilda effect, women in STEM

## Abstract

Despite the widespread perception of computational sciences as objective and merit-based fields, bioinformatics continues to exhibit persistent gender inequalities. This opinion article examines how structural disparities manifest through phenomena such as the citation gap, the Matilda Effect, and the “leaky pipeline,” as well as through sociocultural barriers that shape career progression and scientific recognition. We also discuss the risk of gender bias being reproduced through computational tools, datasets, and algorithmic frameworks developed within the field itself. We argue that sustained institutional and community-level intervention, rather than individual effort, is the only viable path toward a truly equitable scientific community.

## 1 Introduction: the myth of objectivity

Science is frequently idealized as the ultimate meritocracy, a domain where data reigns supreme and professional recognition is granted solely based on the rigor of discovery. However, at the intersection of biology, computation, and statistics, bioinformatics continues to operate under social structures that contradict this ideal. The gender gap in the field is not merely a matter of under-representation; it is a systemic issue where academic worth is often filtered through the lens of gender. UNESCO data showing that women represent less than 30% of researchers worldwide suggest that this filtering begins well before publication, compromising the very objectivity the discipline claims to uphold (Women in science).

The consequences of this inequity may extend beyond individual careers. When gender bias shapes who publishes, who is cited, and who leads research groups, it likely also influences the questions asked and the datasets built—with potential implications for the quality and inclusivity of the science itself, a dynamic supported by evidence that gender-diverse teams tend to produce higher-quality and more innovative research outcomes (Nielsen et al., 2017). This article traces that chain from cultural bias to scientific output.

## 2 The quantitative gap: authorship and citation disparities

The gender gap in bioinformatics is not simply a reflection of broader STEM trends; it is measurably worse than in its neighboring disciplines, and that contrast is itself revealing. In the biosciences, over half of PhD recipients are women; in computer science, that figure drops below 20% (Bonham and Stefan, 2017). Computational biology and bioinformatics fall in between, but closer to computer science than to biology. An analysis of authorship data from PubMed and arXiv confirmed this: journals with “Bioinformatics,” “Computational,” or “Systems” in their titles consistently showed the lowest female representation in the dataset, with women holding an even smaller share of first- and last-author positions than in biology as a whole (Bonham and Stefan, 2017). This gradient is not coincidental: it tracks the degree to which a discipline is perceived as computational and therefore, culturally, as masculine. Notably, the same study found that a female last author significantly increases the likelihood that other authors on the paper are also female, suggesting that structural interventions supporting women’s access to leadership positions would have measurable downstream effects on representation across the field (Bonham and Stefan, 2017).

The bibliometric reality of bioinformatics is sobering. Data on female authorship trends between 2005 and 2017 reveal that representation barely crossed the 35% mark (Saldivar et al., 2020). A 2024 editorial by Martínez-Flores et al. speaks to the consequences of this stagnation: even the work that women do produce tends to reach the community with less visibility and recognition than it merits, depriving the field of perspectives essential to solving complex biological problems (Martínez-Flores et al., 2024).

This pattern is particularly striking in the Latin American context, where women represent approximately 45% of the scientific workforce, a level of numerical parity that might suggest equity, yet remain systematically underrepresented in scientific publishing and receive fewer citations than male colleagues, even when controlling for productivity (López-Bassols et al., 2018; Pradier et al., 2025). The gap between workforce participation and academic visibility suggests that the barriers women face in the region operate less at the entry point than at the stages of recognition, dissemination, and career advancement.

The critical issue extends beyond publication volume to the broader issue of scientific visibility and recognition. A cross-sectional study published in JAMA Network Open found that among more than 10,000 highly cited researchers in biomedicine from 2014 to 2020, only 16% were women compared to 84% men (Shamsi et al., 2022). The COVID-19 pandemic offered a natural stress test of the structural fragility of women’s scientific visibility: an analysis of 1,893 COVID-19 medical papers found that the proportion of women as first authors was 19% lower than in papers published in the same journals in 2019, illustrating how systemic crises disproportionately set back women’s scientific visibility (Andersen et al., 2020).

The Matilda Effect, the systematic under-recognition of female scientists, remains a pervasive force (Rossiter, 1993). A large-scale bibliometric study analyzing 21,509 highly cited authors across 191 disciplines found that women represent only 0.25 researchers per man among the most cited scholars globally, and show lower h-indexes than men in most fields (Goyanes et al., 2025). Crucially, when controlling for productivity and career length, women in natural and exact sciences are actually cited more than their male peers, indicating that the gap reflects structural barriers to visibility and career continuity rather than differences in scientific quality (Goyanes et al., 2025). This ‘citation gap’ does not reflect a divergence in technical quality or methodological rigor; it reveals an entrenched structure where recognition is distributed along gender lines—one that operates across contexts, including in Latin America, where female researchers in STEM have been shown to face lower probabilities of receiving public research funding on their first grant application than their male counterparts, even after controlling for relevant variables (Fiorentin et al., 2022).

## 3 The invisible burden: perception, over-performance, and the leaky pipeline

Beyond h-indexes and rankings, there is a dimension of inequality rarely captured in standard bibliometrics: the constant requirement for over-performance. In a prevailing “culture of doubt”, we argue that women in bioinformatics often face a cumulative cognitive and emotional burden stemming from the need to demonstrate exceptional competence to receive the baseline level of respect that male colleagues receive by default.

This exclusionary environment raises critical questions about the composition of bioinformatics laboratories: Is the male dominance of specific labs a reflection of “talent” or the result of biased evaluation processes? To what extent does the reputation of a male-dominated group deter high-potential female applicants? In a landmark experiment, Moss-Racusin et al. (2012) presented 127 science faculty members at U.S. research universities with identical application materials for a laboratory manager position (differing only in whether the candidate’s name was male or female) and found that the female candidate was consistently rated as significantly less competent and hireable, and was offered a lower starting salary. In bioinformatics, the struggle is twofold: one must not only navigate complex code and biological systems but also confront the implicit presumption of lower technical competence (Moss-Racusin et al., 2012).

Part of this perceived inadequacy is projected not only onto the researcher, but onto the work itself. Bioinformatic analysis is frequently framed as a technical service (something to be requested and delivered) rather than as an intellectual process involving experimental design, methodological choices, statistical reasoning, and iterative interpretation (Bartlett et al., 2017; Dragon et al., 2020; Yanai and Chmielnicki, 2017). This perception is rarely expressed explicitly as gender bias; the colleague requesting “just a quick analysis” may not recognize the implicit devaluation embedded in that assumption. Yet the consequences are not minor. Contributions perceived primarily as technical support are more likely to be minimized in authorship decisions, acknowledgments, funding evaluations, and citation practices. In this sense, we suggest that the citation gap documented in Section 2 may partly emerge, downstream of broader structural devaluation of computational and interdisciplinary labor. When combined with the Matilda Effect, this dynamic produces a self-reinforcing cycle in which both the contribution and the scientist become progressively less visible within the academic record.

Compounding these pressures is the disproportionate allocation of administrative and coordination work, which scholars have termed “academic housekeeping”. Research drawing on data from more than 140 institutions found that female faculty perform significantly more service than their male counterparts, even after controlling for rank, field, and ethnicity, with the gender gap particularly pronounced in STEM fields, where women take on an average of three additional service activities per year (Guarino and Borden, 2017). In bioinformatics, this dynamic is amplified by the discipline’s position as an interface between experimental, clinical, and computational groups: the bioinformatician is frequently called upon to coordinate, integrate, and translate—roles that are essential for projects to function but that generate little formal visibility. The time invested in making a collaboration work does not appear in an authorship line; it appears as a deficit in time available for independent research. This invisibilization of coordination labor is not incidental; it is a structural mechanism through which productivity gaps between men and women are produced and maintained.

A further and underacknowledged dimension of this burden is the penalty associated with interdisciplinarity, a paradox that falls disproportionately on women. Bioinformatics is, by definition, an interdisciplinary field: it demands simultaneous expertise in biology, statistics, and computational science. Yet researchers who operate across disciplinary boundaries frequently face structural disadvantages when evaluated for hiring, promotion, or tenure, as committees anchored in a single discipline struggle to assess work that does not conform to its conventions (Siedlok and Hibbert, 2014). This penalty is not gender-neutral: a recent preprint suggests that women PhD graduates exhibit higher interdisciplinarity on average than their male peers, and that institutional preferences for disciplinary specialization may disproportionately disadvantage them in faculty placement (Zheng et al., 2025). For women in bioinformatics, this creates a form of double epistemic burden: they are expected to demonstrate mastery across multiple domains, while the very breadth of that mastery is used against them in evaluation.

These pressures contribute to what researchers call the “leaky pipeline”, the gradual and disproportionate loss of women from academic and professional trajectories as seniority increases. While women represent nearly half of all doctoral candidates, they account for only 30% of researchers, hold just 20% of full professorships, and lead only 20% of research organizations in Europe (European Commission: Directorate-General for Research and Innovation, 2021). The pipeline does not simply “leak” passively; rather, cumulative structural barriers systematically increase attrition at each career stage. Each stage -from doctoral programs to postdoctoral positions, group leadership, and editorial boards-presents compounding barriers that individually appear manageable but cumulatively produce the representation deficits seen at the top of the field (European Commission: Directorate-General for Research and Innovation, 2021). This dynamic has been documented across Latin America: in Colombia, the leaky pipeline effect has been confirmed across career stages, with women progressively underrepresented as seniority increases and particularly in STEM disciplines (López-Aguirre, 2019). In Argentina specifically, even after controlling for academic productivity and career stage, female researchers in STEM show a 6.2 percentage point lower probability of receiving public research funding on their first grant application than male counterparts, and that gap widens to 8 percentage points among senior researchers, illustrating how structural disadvantage compounds over time (Fiorentin et al., 2022).

## 4 Spaces of resistance: the necessity of specialized networks

The emergence of gender-specific initiatives, such as Women in Bioinformatics and Data Science Latin America (WBDS LA), is frequently met with questions regarding their necessity. The answer lies in the historical and institutional structure of the field, in which informal mentorship networks, hiring practices, and conference cultures have favored male participation and visibility (Pradier et al., 2025; Rueda et al., 2022). The creation of WBDS LA in 2019 responded directly to this regional reality. Its first conference, held in 2020, brought together women researchers from across Latin America working in bioinformatics and data science, generating a space for collaboration and visibility that institutional channels had failed to provide (Jiménez et al., 2021). The need for such spaces reflects a structural gap: quantitative studies show that women scientists in the region publish less and have fewer opportunities to participate in international collaborations, with differences that are more pronounced depending on the country (López-Bassols et al., 2018).

These organizations are not mechanisms of exclusion; they are essential tools for survival and visibility. They provide the collaborative networks that traditional institutional systems often fail to offer. A concrete example is the Bioinfo4Women program at the Barcelona Supercomputing Center, which since 2022 has worked not only to support career development but also to raise awareness of how gender bias propagates through AI applications and biomedical research datasets—promoting the use of more representative data and methodologies to mitigate bias at the research design stage (Network, 2025). This dual focus, on people and on the science they produce, represents the most complete model of gender equity intervention in the field.

While “safe spaces” should not be a prerequisite for professional fulfillment, they remain a powerful tool for change as long as academia penalizes motherhood (Fiorentin et al., 2022), overlooks female citations (Goyanes et al., 2025), and maintains hiring biases (Moss-Racusin et al., 2012).

## 5 Beyond representation: when gender bias becomes scientific bias

The consequences of gender inequity in bioinformatics do not stop at individual careers—they extend into the science itself. When research teams lack diversity, the questions they ask, the populations they study, and the datasets they build may reflect that homogeneity (Nielsen et al., 2017). In a field that produces computational tools, predictive models, and genomic analyses used in clinical and public health contexts, this is not a peripheral concern: it is a validity problem.

Algorithms trained on datasets that underrepresent women, or developed within scientific cultures lacking diverse perspectives, risk producing outputs that perform unequally across populations. This problem has already been documented across multiple areas of biomedicine and artificial intelligence, where biased training datasets can reduce predictive accuracy and clinical relevance for underrepresented groups (Cirillo et al., 2020). In bioinformatics, similar risks emerge in the construction of genomic reference datasets, benchmarking frameworks, annotation practices, and AI-assisted analytical tools. A 2024 preprint analyzing 211 datasets in the Gene Expression Omnibus (GEO) found biases in gender and ethnicity representation, highlighting how data infrastructure in the field may reflect and amplify the demographic gaps of the teams that produce it (Gondal, 2024). This pattern extends across multiple layers of bioinformatic practice: standard pipelines have historically excluded or masked sex-linked genomic regions, resulting in poorer variant calling for female-derived samples (Pinto et al., 2023); sex metadata labels are absent in the majority of publicly available gene expression datasets, with female samples underrepresented in drug studies with direct implications for reproducibility (Flynn et al., 2021); and polygenic risk scores trained on ancestry-skewed cohorts show reduced predictive accuracy when applied to underrepresented groups, whether defined by sex, ethnicity, or geographic origin - raising broader concerns about equity and generalizability in clinical genomic applications (Sirugo et al., 2019).

The argument for gender equity in bioinformatics is therefore not solely ethical; it is epistemological. We argue that diversity influences not only who participates in science, but also which biological questions are prioritized, which assumptions remain unchallenged, and which forms of expertise are recognized as intellectually valuable (Nielsen et al., 2017). A field that systematically marginalizes a substantial portion of the scientific talent pool does not simply lose potential; it risks producing less robust and less generalizable science. Addressing the culture of devaluation is therefore inseparable from improving the rigor, reproducibility, and inclusivity of the field itself. Although the structural barriers documented here are not unique to bioinformatics, the interdisciplinary and data-driven nature of the field makes these challenges particularly visible—and particularly consequential.

## 6 Discussion: a collective responsibility for structural change

Recent years have seen meaningful progress: the emergence of gender-focused networks such as WBDS LA, institutional programs, and dedicated publication venues including this Research Topic represent concrete advances in visibility and community-building. Nevertheless, significant gaps remain, particularly in the systematic study of gender bias within bioinformatic datasets and tools, and in the development of standardized equity metrics for peer review and hiring processes. Future work should prioritize these areas, as well as longitudinal studies tracking the leaky pipeline specifically within computational biology careers.

This perspective is written from within the Latin American scientific community, a context that adds particular urgency to the structural challenges described above. Despite achieving near-parity in overall researcher numbers, with women representing approximately 45% of the scientific workforce across the region, Latin American women remain systematically underrepresented in scholarly communication, with evidence showing they tend to publish within regional academic circuits while men dominate global ones, compounding their invisibility in international citation networks (Pradier et al., 2025). The cases of Argentina and Uruguay are particularly illustrative: both countries have achieved numerical parity among researchers (Women in science), yet this parity does not translate into equivalent representation in publications, citations, or leadership. In Uruguay, even at a public university with no formal access barriers, women remain underrepresented in STEM career selection and persistence (Bruzzese et al., 2025), suggesting that structural barriers operate independently of formal access, and that headcount parity is a necessary but insufficient measure of equity. Regional data further document horizontal segregation across STEM disciplines and persistent vertical barriers: in Argentina, female researchers face lower probabilities of receiving public funding at every stage of grant application (Fiorentin et al., 2022); in Colombia, the leaky pipeline has been confirmed across career stages (López-Aguirre, 2019); and across eleven countries in the region, family pressures, stereotypes, lack of mentors, and institutional inertia have been identified as compounding factors that no single intervention can address in isolation (López-Bassols et al., 2018). These patterns suggest that addressing gender inequity in bioinformatics (and in other disciplines) requires not only global structural interventions but also region-sensitive policies that account for the specific contexts of Latin American scientific systems.

The path forward cannot rely on the expectation that women simply “work harder” to bridge the gap. Equity requires active, structural intervention:

- Bias Audits: Drawing on evidence of persistent gender gaps in citation and recognition patterns (Goyanes et al., 2025), we propose that editorial boards and journals systematically review their peer-review processes to identify and address gender bias in authorship and citation patterns
- Conscious Citation: Given evidence that implicit bias shapes how researchers evaluate and reference the work of others (Moss-Racusin et al., 2012), we argue that researchers, particularly those in senior positions, should audit their reference lists to avoid overlooking the contributions of female colleagues
- Recognition of Interdisciplinary Work: Building on documented structural disadvantages faced by interdisciplinary researchers in academic evaluation (Siedlok and Hibbert, 2014), we proposed that hiring committees, funding bodies, and tenure evaluators develop criteria adequate to computational and interdisciplinary contributions. Treating bioinformatic analysis as a “technical service” rather than an intellectual contribution reproduces the very structures that fuel the gender gap
- Institutional Accountability: In light of evidence that women in academia carry a disproportionate burden of service and coordination work (Guarino and Borden, 2017), we argue that zero-tolerance policies for harassment and formalized mentorship structures support diverse career trajectories at every stage of the pipeline.

The future of bioinformatics must be diverse, not to satisfy a metric or quota, but because high-quality science cannot afford to marginalize fifty percent of the global talent pool. Until the culture of devaluation is addressed -in hiring, in citation, in recognition, and in the design of the science itself-the “objectivity” of bioinformatics remains a myth.

## Data Availability

The original contributions presented in the study are included in the article/supplementary material, further inquiries can be directed to the corresponding author.
